# MRI-Based Personalized Transcranial Direct Current Stimulation to Enhance the Upper Limb Function in Patients with Stroke: Study Protocol for a Double-Blind Randomized Controlled Trial

**DOI:** 10.3390/brainsci12121673

**Published:** 2022-12-05

**Authors:** Yeun Jie Yoo, Hye Jung Park, Tae Yeong Kim, Mi-Jeong Yoon, Hyun-Mi Oh, Yoon Jung Lee, Bo Young Hong, Donghyeon Kim, Tae-Woo Kim, Seong Hoon Lim

**Affiliations:** 1 Department of Rehabilitation Medicine, St. Vincent’s Hospital, College of Medicine, The Catholic University of Korea, Seoul 16247, Republic of Korea; 2 Department of Rehabilitation Medicine, National Traffic Injury Rehabilitation Hospital, Yangpyeong 12564, Republic of Korea; 3 Research Institute, NEUROPHET Inc., Seoul 06234, Republic of Korea

**Keywords:** transcranial electrical stimulation, transcranial direct current stimulation, non-invasive brain stimulation, stroke, upper limb, neuromodulation, neurorehabilitation

## Abstract

Transcranial direct current stimulation (tDCS) has been shown to have the potential to improve the motor recovery of the affected upper limbs in patients with stroke, and recently, several optimized tDCS methods have been proposed to magnify its effectiveness. This study aims to determine the effectiveness of personalized tDCS using brain MRI-based electrical field simulation and optimization, to enhance motor recovery of the upper limbs in the patients. This trial is a double-blind, randomized controlled trial in the subacute to chronic rehabilitation phase. Forty-two adult stroke patients with unilateral upper limb involvement will be randomly allocated to three groups: (1) personalized tDCS with MRI-based electrical field simulation and optimized stimulation, (2) conventional tDCS with bihemispheric stimulation of the primary motor cortex, and (3) sham tDCS. All three groups will undergo 10 intervention sessions with 30 min of 2-mA intensity stimulation, during a regular upper limb rehabilitation program over two weeks. The primary outcome measure for the motor recovery of the upper limb impairment is the Fugl–Meyer assessment for the upper extremity score at the end of the intervention, and the secondary measures include changes in the motor evoked potentials, the frequency power and coherence of the electroencephalography, performance in activities of daily living, and adverse events with a 1-month follow-up assessment. The primary outcome will be analyzed on the intention-to-treat principle. There is a paucity of studies regarding the effectiveness of personalized and optimized tDCS that considers individual brain lesions and electrical field characteristics in the real world. No feasibility or pivotal studies have been performed in stroke patients using brain MRI, to determine a lesion-specific tDCS simulation and optimization that considers obstacles in the segmentation and analysis of the affected brain tissue, such as ischemic and hemorrhagic lesions. This trial will contribute to addressing the effectiveness and safety of personalized tDCS, using brain MRI-based electrical field simulation and optimization, to enhance the motor recovery of the upper limbs in patients with stroke.

## 1. Introduction

Many patients complain of upper limb paralysis, which remains, in many cases, as a disability that interferes with the independence of daily living [1]. These motor impairments of the upper limbs would be known to relate to the corticospinal tract (CST) integrity, originating from the primary motor cortex [2,3]. In addition, the cerebellar afferent and efferent tracts also contribute to the motor function of the upper limbs [4,5]. Combining this knowledge for the anatomical basis of the upper limb motor function, the primary motor cortex and CST are the most critical structures for the motor function of the upper limbs. Thus, previous studies for noninvasive neuromodulation via repetitive transcranial magnetic stimulation (rTMS) or transcranial direct current stimulation (tDCS), have targeted the ipsilesional primary motor cortex for enhancing the motor function of the upper limbs [6,7,8].

Proper treatment and effective recovery in patients with a stroke are important issues for the patients themselves, their families, neurologists, therapists and physiotherapists. Many stroke survivors have ongoing impairments of the upper limbs [9]. Recently, noninvasive brain stimulation has been introduced to reduce the impairments of the upper limbs [8,10,11]. Among the various types of noninvasive brain stimulation, transcranial direct current stimulation (tDCS) is being actively studied [12,13]. The tDCS has recently received the most attention because of its safety, convenience, and portability [12,14,15] A constant, low-intensity current (1 to 2 mA) is passed through two electrodes placed over the head and modulates the neuronal activity [16,17]. The widespread opinion is that anodal stimulation increases the cortical excitability, whereas cathodal stimulation reduces the cortical excitability (by hyperpolarization and depolarization, respectively) [18].

The level of evidence for tDCS is still low because a standardized protocol has not been fully established [19]. Particularly, this is because the results of studies that tried to verify the tDCS effects were inconsistent [20], and a main factor was the large variation in the treatment effect between individuals, even when the same protocol was applied [21]. High levels of interindividual variations in the tDCS effects are thought to be because the low-intensity current transmitted through the scalp is influenced by the brain structure, such as the characteristics of brain anatomy [22]. As a result, these inconsistent findings have been taken as a lack of evidence for its effectiveness [23,24]. Although a 10–20 EEG guide was introduced to apply tDCS electrodes in a manner that considers the variation in individuals’ head geometry, the guide did not provide an exact match for the patients’ brains in clinical neurorehabilitation settings.

Recently, some studies have attempted to define the optimized stimulation parameters and to reveal the mechanisms of interaction between the electric field (E-field) induced by tDCS and the cortical neurons and their therapeutic effects [22]. The lack of an analytical and technical pipeline enabling the optimization of stimulation parameters across individuals is a factor that interferes with the accumulation of evidence. Optimization methods requiring the analysis of individual MR images to make a brain model and the calculation of E-fields induced by tDCS, is very labor intensive, and there is an inconvenience in using multiple types of research software. These reasons have blocked the application of tDCS with the calculated E-fields in the clinical setting. Thus, we recently developed a method that analyzes the magnetic resonance image of an individual, to generate a brain model and then calculates the magnitude of the electric field generated by tDCS, through the analysis of the brain structure, in a single software package [25]. To maximize the effect of tDCS, not only the individual brain structures but also the stroke lesions must be considered [26], so the software was developed to include methods that could segment the stroke lesions and calculate the conductivity of the stroke lesions. One previous study reported that the optimized electrode montages which applied MRI-based individual head models for tDCS, could yield the maximal stimulation target, compared to conventional montages [27]. However, the study has no results about the clinical effect.

Taking into account the mentioned limitations of the conventional tDCS method and the results of a previous study, our ongoing development of optimized software, and current clinical demands, we hypothesized that MRI-based optimized personalized tDCS would be better than conventional tDCS, to treat patients with a stroke for improving the upper limb function. Thus, we will investigate the efficacy of the MRI-based optimized personalized tDCS for improving the upper limb function in patients with a subacute and chronic stroke via a multicenter prospective randomized double-blinded controlled trial. We present the rationale and precise methods from our planned trial: MRI-based personalized transcranial direct current stimulation to enhance the upper limb function in patients with a stroke: A randomized double-blinded controlled trial. The proposed protocol seeks to establish the benefits from personalized anodal tDCS with two control groups, a sham control group and a conventional anodal tDCS group, to prove the treatment efficacy of the upper limb motor function in patients with a stroke.

## 2. Materials and Methods

### 2.1. Trial Design

**Figure 1:**
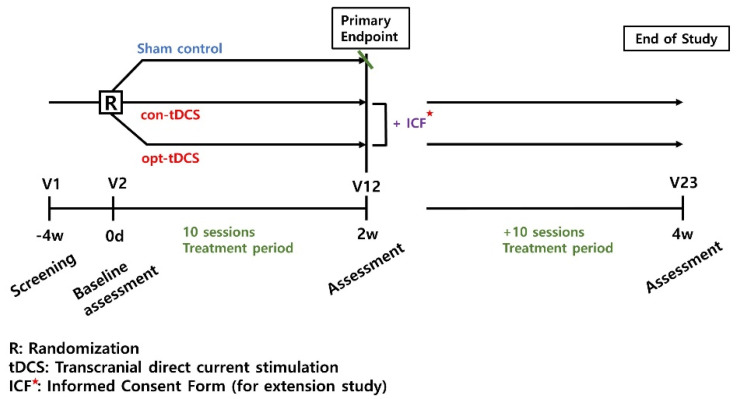
Study design.

This trial design is a prospective randomized placebo and actively controlled (using sham-tDCS and conventional-tDCS), a parallel group, double-blind, multicenter, phase 2 feasibility study (Figure 1). A personally optimized tDCS (opt-tDCS) will be compared to the sham-tDCS or conventional-tDCS (con-tDCS) in patients with a subacute to chronic stroke. All interventions are performed with the usual rehabilitation, such as occupational therapy, physical therapy, and peripheral functional electrical stimulation.

### 2.2. Participants Involvement and Ethics Approval

This protocol was approved by the Korean Ministry of Food and Drug Safety and complied with the ethical standards, based on the Declaration of Helsinki. The final protocol was approved by the Ethics Review Boards of the Catholic University (approval number: VC21DIDS0085) and the National Traffic Injury Rehabilitation Hospital (approval number: NTRH-21004). Written informed consent will be obtained from each participant.

### 2.3. Recruitment

Participants will be screened and recruited from two hospitals: The Catholic University of Korea, St. Vincent’s Hospital and the National Traffic Injury Rehabilitation Hospital in Republic of Korea.

### 2.4. Inclusion and Exclusion Criteria

The *inclusion criteria* are as follows: (1) age over 19 years old; (2) stroke confirmed by brain MR images; (3) first-onset stroke; (4) subacute or chronic phase (at least 4 weeks after the stroke onset); (5) unilateral upper limb paralysis; (6) an upper extremity FMA score (out of 66) of 56 or less; and (7) capacity to understand the explanation of this clinical trial, voluntary participation and written agreement to abide by the precautions.

The exclusion criteria will be the following: (1) those with a disease that may affect the function of the paralyzed arm, such as recurrent stroke, traumatic brain injury, spinal cord injury, degenerative brain disease, such as Parkinson’s disease, or upper extremity nerve injury; (2) those who cannot conduct clinical trials according to the instructions, due to cognitive decline; (3) those who have severe communication difficulties, due to total aphasia; (4) those with evidence of delirium, confusion or other disturbances of consciousness; (5) those with an uncontrolled medical or surgical disease; (6) those who cannot undergo tDCS due to scalp disease; (7) those who have metallic substances (e.g., clip, coil, or metabolic foreign body) in the brain; (8) those with pacemakers or cochlear implants; (9) those who have used stimulation devices that are similar to the medical devices used in this clinical trial, within at least 1 year, or have experience in participating in related clinical trials; (10) those with severe neurological disorders accompanying major psychiatric disorders; (11) those with a history of epilepsy; (12) those who have medical contraindications to brain imaging acquisition; (13) those who have taken prohibited concomitant medications or need to change medications that may affect their cognitive and motor function changes, through changes in brain activation during the study period (however, this does not apply to those who are taking the drug in a stable manner); and (14) pregnant or lactating women and those who plan to become pregnant during the clinical trial period.

### 2.5. Enrollment and Randomization

Participants who meet all the inclusion criteria and none of the exclusion criteria and have given informed consent, will be evaluated by the investigators for enrollment. At enrollment, the information will be collected, including the demographic data, body measurements, stroke history, complications, and baseline function measures. The investigator will verify that the patient is eligible to participate and enter information into the electric data capture and web allocation system, as required.

The participants will be randomly assigned to a personally optimized tDCS group (opt-tDCS), a conventional tDCS group (con-tDCS; an active control group), or a control group (sham-tDCS) in a 1:1:1 ratio, in the order of registration. To minimize the effects, based on the disease severity, a Fugl–Meyer assessment for the upper extremities (FMA-UE) cutoff values were used to divide patients into 0–34 (moderate to severe) and 35–56 (mild to moderate) groups, and the onset period was used to stratify patients into two groups, subacute (onset 1–6 months) and chronic (greater than 6 months). Statisticians who were not otherwise involved in this clinical trial used statistical software to randomize the randomization numbers, according to the block randomization method. The issued randomization number was put in a randomization bag and delivered to the medical device manager of each site. The allocation sequence was concealed from an independent researcher. A sealed envelope was given to the coordinator of the physical therapy area to identify the group for each participant.

### 2.6. tDCS Treatment, Simulation and Blinding

#### 2.6.1. tDCS Device

**Figure 2:**
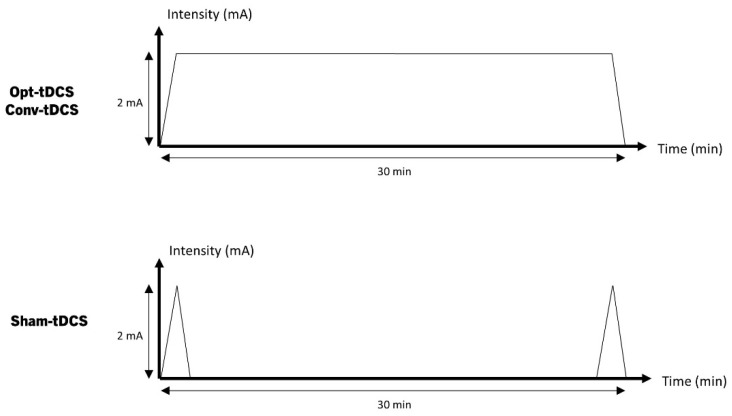
tDCS and sham tDCS protocol.

**Figure 3:**
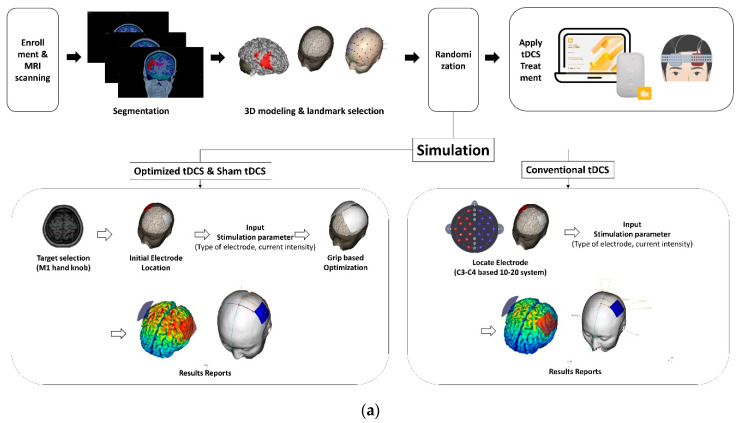

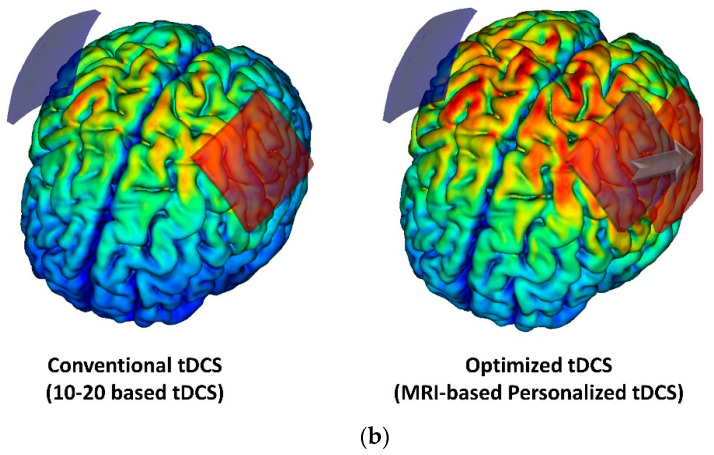
(**a**) The intervention methods for the three groups. (**b**) Computational results of the optimized and conventional tDCS simulation.

Participants will undergo an intervention tDCS treatment using a battery-driven and portable device (Neurophet innk, Neurophet, Seoul, Republic of Korea), programmed to deliver either active or sham tDCS. The device delivers 2.0 mA (with 30 s for ramping up and ramping down; Figure 2) of the direct current through the two sponge-coated electrodes (electrode size: 5 × 5 cm^2^). The tDCS device used in this study can check the impedance values occurring in patient’s skin, in real time, during all tDCS sessions. If an impedance above 13 kOhm occurs, the stimulation is stopped, and the medical device investigator confirms the patient’s condition and skin. This may reduce the adverse events that may occur on the patient’s skin, due to the increased impedance. In the sham-tDCS condition, the device delivers 120 s of the current, including 30 s for ramping at the beginning and end of each session, to maintain the blinding procedure. The tDCS treatment was performed 10 times over a total of 2 weeks, and sham-tDCS was also applied 10 times with sham stimulation for 2 weeks. Following this duration, both opt-tDCS and con-tDCS groups, but not the sham-tDCS group, will receive the opportunity to provide additional written consent for an extension study; if the participants give consent, the extension study will be conducted for two additional weeks. During the 2 or 4 weeks of intervention, if there was a usual rehabilitation program that was being performed before participation in this study, the program and frequency could be continued without any changes. The detailed intervention methods regarding tDCS for each group, is as follows (Figure 3a). The representative figure which represents the difference between optimized tDCS and conventional tDCS simulation results in participant is Figure 3b.

#### 2.6.2. tDCS Simulation

The GUI based Software ‘Neurophet tES LAB 3.0’ that can calculate the tDCS-induced electrical fields after considering an individual’s brain structure by analyzing and segmenting the T1-weighted MR image of the individual participant, is applied in this trial to plan the tDCS protocol. Following enrollment, each participant’s T1-weighted MR images, taken at the baseline, are analyzed in the software, of the skin, skull, brain tissues (cerebral gray and white matter, cerebellum gray and white matter, cerebrospinal fluid, ventricle) and the affected tissue; a region affected by stroke and not fully replaced with CSF, are segmented in the software, based on an electrical conductivity. At this time, for the segmentation of the whole brain tissue, including the stroke-affected tissue, a U-Net structure, i.e., an encoder-decoder based network, is used. Given a T1-weighted MR image as input to the network, the encoder first extracts the feature from the input image. The features are then provided to a decoder network, and during the decoding process, the decoder network reconstructs the features to the original image size [28]. Then, the investigators could modify the results of the tissue segmentation, including the affected tissue. We set the electrical conductivity of eight tissues as follows: skin, 0.465; skull, 0.010; cerebral and cerebellar gray matter, 0.265; cerebral and cerebellar white matter, 0.126; CSF and ventricle, 1.65; and affected tissue 0.08089. The principal investigators will confirm the segmented results, and all procedures are applied to all participants, according to the same rules. Following segmentation, a 3D brain model of each participant is created. An investigator selects the landmarks, including nasion, inion and the right and left preauricular points in the model. The next steps are continued, based on the randomized groups.

##### Opt-tDCS Group (Personally Optimized tDCS; Experimental Group)

The investigator selects a target that represents the structural M1 representation of the hand in the cerebral gray matter of the participant’s hemisphere with the lesion. Next, the investigator attaches the anode electrode directly above the target, and the cathode electrode at the position representing M1 in the contralesional hemisphere. These electrode locations are the initial locations for the tDCS optimization calculations. Based on the locations, the two electrode locations that can maximally stimulate the M1 target in the frontal area of the participant’s ipsilesional and contralesional hemispheres are chosen. At this time, the investigators input the tDCS simulation parameters, such as the current intensity (2 mA), electrode type (5 × 5 square type), and conductivity of the brain tissue, to calculate the optimal electrode locations. The volumes of all segmented layers generated the 3D brain model, and the simulation calculated the magnitude of the electric field generated by tDCS, through the brain structure. By applying the optimized tDCS simulation to the patients, the simulation finds the combination of the tDCS electrodes to generate the maximum electric fields or the maximum inward electric field values in the region of interest. Among the several location candidates, when the combination of the electrode locations that can maximally stimulate the target is calculated, medical device investigators find the corresponding electrode position, based on their landmarks and start the tDCS.

##### Con-tDCS Group (Conventional tDCS Based on a 10–20 EEG System.; Active Control Group)

As with the opt-tDCS group, the investigators created a 3D brain model for each participant and selected the landmarks for the tDCS electrode location generation in tES LAB software. Then, the software automatically generates the locations, according to a 10–20 EEG-based system in the software. For this group, the electrode positions are selected, according to the 10–20 EEG system, an anode electrode is attached to the ipsilesional side (C3 or C4), and a cathode electrode is attached to the contralesional side.

##### Sham-tDCS Group

Simulations for the sham-tDCS group were performed with the same procedure as for the opt-tDCS group; therefore, the electrode locations were the same as that group, but during the tDCS stimulation, the ‘sham mode’ was applied for the initial and final 1-min periods with a ramping up and down for 30 s.

### 2.7. Blinding

In the case of this study, it is difficult to keep the examiner blinded because the location of the tDCS electrodes is different, depending on the treatment method. Therefore, an unblind investigator, in charge of the application of medical devices for clinical trials, is separately assigned to each site, to maintain the blindness of the blinder. The unblinded investigator does not participate in the research process other than the treatment to minimize the impact on the efficacy and safety evaluation of the investigator, due to an unblinded design.

### 2.8. Primary Outcome

Upper limb functional changes will be evaluated by the FMA-UE scores, between the baseline and postintervention, at 2 weeks. The FMA-UE is a performance-based impairment index, including the motor function with a range of motion and sensation in patients with a stroke, and the results are represented as a numerical score with a maximum of 66 points [27,29].

### 2.9. Secondary Outcomes

The secondary outcomes include the changes in the motor evoked potentials (MEPs); frequency power and coherence of electroencephalography (EEG); upper extremity functions; Wolf motor function test (WMFT) [29,30], box and block test (BBT) [31], performance in activities of daily living (ADLs) [32], and adverse events with the 1-month follow-up assessment (for details, see Table 1).

### 2.10. Sample Size Estimates

Most studies that aimed to assess the efficacy of the MRI-driven optimized tDCS, were retrospective computational studies or case reports [26]. Moreover, this trial is the first to report the efficacy of the optimized tDCS on the upper limb functions in stroke patients. Considering that there were no prior data on the effects of the optimized tDCS, using an MRI-driven tDCS-induced electrical field calculation simulation on stroke patients, we estimated that enrolling 42 patients, considering a dropout rate of 10% for each group, would be a reasonable approach for an exploratory trial.

### 2.11. Statistical Analyses

The descriptive statistics (average, standard deviation, median, minimum, and maximum) are presented for each group for the change in the primary outcomes at 2 weeks, compared to the baseline (0 weeks), and the comparison of differences between the groups will be the repeated measures ANCOVA that considers time as a covariate. If statistically significant changes were confirmed in the comparison of the differences between the three groups, a repeated ANCOVA that considered the timing of the differences between the two groups (e.g., opt-tDCS and con-tDCS, opt-tDCS and sham-tDCS) was planned as a post hoc analysis. The results corresponding to the secondary outcomes will be presented as descriptive statistics (frequency, ratio) for each group, based on the change in score at 4 weeks, and the difference between the two groups, will be analyzed using the repeated measures ANCOVA, that considers time.

The primary outcome will be analyzed in the intention-to-treat population, and the safety outcomes will be analyzed in the per-protocol population. No protocol interim analysis is planned, and a total of 42 patients will be enrolled in this clinical trial over a total of 30 months. This sample will be able to evaluate the clinical applicability of the optimized tDCS treatment in the subacute to chronic stages of a stroke and aims to verify the trends in the intergroup differences and the changes to demonstrate the statistically significant differences in the upper limb function improvement between the two groups.

The outcome analysis of this trial will be largely divided into a safety set (SS) group, a full analysis set (FAS) group, and a per-protocol set (PP) group. The efficacy evaluation will analyze the PP group as the main analysis group and analyze the FAS group. If the results are different across the analysis groups, the results of each analysis method will be presented and analyzed to identify the reason. The safety assessment will be carried out in the SS group, and the significance level will be set at 0.05.

### 2.12. Safety, Protocol, and Data Monitoring

The potential tDCS adverse events and side effects that may occur to the participants during the treatments are identified during stimulation as well as up to 30 min after the end of stimulation. If adverse events occur, they will be diagnosed by a study physician and will be reported to IRBs, according to the criteria of severity (mild, moderate, and severe). If the events are severe, the investigators must report that to the Korean Ministry of Food and Drug Safety, which will advise whether to continue, modify or stop the intervention and decide whether to unmask the group assignment.

## 3. Discussion

This prospective multicenter double-blind controlled trial aims to prove that the patient’s own MRI-based optimized tDCS treatment beneficially impacts the improvements in the upper limb function and the parameters related to ADLs or neurophysiology in patients with a stroke. Although positive results for the tDCS effectiveness has been reported by several studies, the level of clinical evidence and practical use in the clinical settings are still limited [24,25]. Several studies evaluating the tDCS effectiveness have shown inconsistent results for improving the upper limb function in patients with a stroke [14,33,34], and the reasons inferred by researchers for the limited effectiveness are as follows: (1) there is a variety in the tDCS stimulation montage and parameters used; and (2) the personalized optimization of tDCS has not been applied. Therefore, recent studies have sought ways to overcome individual variation in the tDCS treatment effects.

This challenge contributes to previous studies performing randomized controlled trials with a large sample size; in addition, these studies have suggested to conduct dose-controlled tDCS studies with MR images, based on head models [35]. A previous study created computational models with MR images of two chronic stroke patients, analyzed the distribution of the TMS and tDCS-induced electrical fields, and tried to verify that transcranial brain stimulation is a safe method for chronic stroke patients, compared to healthy adults [36]. This report described a computational study on how the location of the lesion and brain atrophy could affect the stimulation-induced electrical fields and effects and intended to provide a basis for the computational studies to use MR image-based head models for clinical application. However, the fact that only two patients were analyzed and the relative positions of the tDCS electrode and TMS coil, compared to the lesion location, were divided into only nine positions, limited this study as a basis for application in clinical patient cases. Another study used the necessary research tools to segment the brain lesions using individual images more accurately and to simulate tDCS in a manner that considered the lesions and reviewed the clinical applicability. In this study, the results were compared and analyzed across each of the 16 conductivity values and considered that the tDCS-induced electrical fields depended on the conductivity of each region of the brain tissue [37].

In this way, previous studies have attempted the strategy of reducing the variations, using individual MRI-based computational models, but they could not actively achieve adequate results because it is not easy to apply procedures including MR image analysis and segmentation, volume conductive head modeling, and stimulation simulation. In particular, the description of the image analysis and segmentation is not yet perfect, and researchers must use several research tools for performing a series of procedures, including MR image segmentation, 3D brain modeling, and stimulation simulation [38]. Such open-source research tools are generally difficult to use, require a long period of time, and above all, they are not easy to apply in clinical fields, due to poor user convenience. Taking this into account, we developed a personally optimized tDCS model with a MRI-based phantom head [25] and developed the software ‘tES LAB’, which can calculate the tDCS-induced electrical fields. tES LAB is GUI-based and has the advantage of being able to perform a series of processes, including MR image segmentation and personally optimized tDCS simulations, within one software package, so it can be used without professional researcher personnel.

However, our study protocol has several limitations. First, the patients in a craniectomy state would be excluded from this study because of unsolved safety issues. Usually, patients with a huge intracranial hemorrhage may be taken for a craniectomy. The distributions of patients slightly differ from the real-world distribution. Second, we conducted tDCS with just 2.0 mA in this study protocol. Recently, 4.0 mA tDCS may stimulate more widely, compared to 2.0 mA [39]. However, the Korea Disease Control and Prevention Agency permitted up to 2 mA for tDCS in the clinical field. Thus, we planned to conduct tDCS with 2 mA in this protocol.

In this study, we will attempt to apply the optimized tDCS treatment that considers the brain structure of individual patients and try to improve treatment in real clinical environments, involving rehabilitation in subacute to chronic periods, compared with a conventional tDCS, which is the most active period of rehabilitation after stroke. If stroke patients, during this period with motor disabilities, can safely undergo tDCS treatment together with parallel treatment regimens, acute or chronic stroke patients after discharge may also be able to use it. Our findings are expected to show that the patient’s optimized tDCS treatment will probably be pragmatic for patient care and contribute to overcoming previously reported limitations on tDCS treatment. In addition, it is expected that the effect of optimized tDCS, which has always been limited only to simulation study results, can be verified from the actual therapeutic point of view.

Trial Status: Recruitment of participants started in November 2011 and will be completed in December 2023. This manuscript reports protocol version 4.1 (25 January 2022).

## Figures and Tables

**Table 1:** Schedule of enrollment, interventions and assessment.

	Screening	Intervention			Outcomes	Extended Intervention		Outcomes
		Baseline						
Visit	V1	V2	V3~V6	V7~V11	V12	V13~V17	V18~V22	V23
ENROLLMENT								
Informed consent	V					V		
Eligibility screen	V	V						
Randomized allocation		V						
INTERVENTIONS								
Opt-tDCS Conv-tDCS Sham-tDCS		V	V	V		V	V	
ASSESSMENTS								
Baseline variables Vital signs Physical examination MRI scan MEPs EEG Functional examinations		V						
Outcome variables MRI scan MEPs EEG Functional examinations		V			V			V
Other variables Adverse events		V	V	V	V	V	V	V

## Data Availability

The data will be presented in this study via the next article after the completion of the double-blind randomized controlled trial.
